# Impact of Quantitative Computed Tomography-Based Analysis of Abdominal Adipose Tissue in Patients with Lymphoma

**DOI:** 10.3390/hematolrep15030049

**Published:** 2023-08-04

**Authors:** Federico Greco, Bruno Beomonte Zobel, Carlo Augusto Mallio

**Affiliations:** 1 Department of Radiology, Cittadella della Salute Azienda Sanitaria Locale di Lecce, Piazza Filippo Bottazzi, 2, 73100 Lecce, Italy; 2 Unit of Diagnostic Imaging and Interventional Radiology, Department of Medicine and Surgery, Università Campus Bio-Medico di Roma, Via Alvaro del Portillo, 21, 00128 Roma, Italy; 3 Fondazione Policlinico Universitario Campus Bio-Medico, Via Alvaro del Portillo, 200, 00128 Roma, Italy

**Keywords:** adipose tissue, body composition, cancer cachexia, computed tomography, fat, hematologic malignancies, lymphoma, sarcopenia, precision medicine, visceral obesity

## Abstract

Quantitative abdominal adipose tissue analysis is important for obtaining information about prognosis and clinical outcomes on a wide array of diseases. In recent years, the effects of abdominal adipose tissue compartments in patients with lymphoma and the changes in their distribution after therapies have been studied. This information could facilitate the improvement of therapies in patients with lymphoma, to prevent or treat both visceral obesity and sarcopenia. Opportunistic analysis of body composition on computed tomography (CT) images might contribute to the improvement of patient management and clinical outcomes together with implementation of targeted patient-tailored therapies. The purpose of this literature review is to describe the role of CT to evaluate abdominal adipose tissue quantity and distribution in patients with lymphoma.

## 1. Introduction

An excessive amount of adipose tissue, defined as overweight or obesity, is associated with an increased risk of morbidity and mortality and is a well-known risk factor for oncological diseases [1]. A meta-analysis of prospective studies (over 1 million subjects) highlighted that body mass index (BMI) is positively associated with an increased risk of developing Hodgkin lymphoma (HL) and non-Hodgkin lymphoma (NHL) and with NHL mortality [2,3,4].

The obesity-induced proinflammatory cytokine storm promotes the proliferation of cancer cells. Indeed, it has been shown that the proinflammatory state due to increased adiposity through hypertrophic adipocytes, with consequent release of protumor adipokines into the bloodstream, can modulate the aggressiveness of Hodgkin Reed–Sternberg lymphoma cells [5]. One of the most important pathways in regulating the obesity-associated proinflammatory response is NF-κB [6]. NF-κB signaling is a hallmark that promotes lymphoma growth and survival by activating anti-apoptotic and pro-proliferative gene programs in many lymphoid malignancies including HL, diffuse large B-cell lymphoma (DLBCL), mucosa-associated lymphoid tissue (MALT) lymphoma, primary effusion lymphoma and adult T-cell lymphoma/leukemia (ATLL) [7,8]. Moreover, the increase in several adipose tissue-released cytokines such as interleukin (IL)-6, IL-8, IL-10, monocyte chemoattractant protein (MCP)-1 and IL-1 have been shown in several types of lymphoma [9].

A conventional widely used obesity metric is BMI. However, this metric cannot distinguish different tissues or separately quantify the two main compartments of abdominal adipose tissue: visceral adipose tissue (VAT) and subcutaneous adipose tissue (SAT) [10]. Adipose tissue is a metabolic/endocrine organ, but, in greater detail, VAT has a greater hormonal and metabolic activity than SAT because these two compartments have a different molecular and cellular composition [11]. Indeed, VAT releases adipokines, proinflammatory cytokines and growth factors related to the development of obesity-related tumors [12,13].

Quantification of adipose tissue compartments in oncologic patients is mainly performed with computed tomography (CT), a fundamental imaging technique for non-invasive tissue evaluation and characterization, which recently gained attention in the context of precision medicine [14,15,16]. This manuscript summarizes the available evidence on CT abdominal adipose tissue compartments quantification in patients with lymphoma, emphasizing the role of abdominal adipose tissue for risk prediction and prognosis assessment.

## 2. Methods

The literature search was performed on February 2023 using MEDLINE PubMed Central and selecting exclusively articles written in English with no time limit. The combination of keywords used was: “adipose tissue lymphoma”, “abdominal fat lymphoma”, “visceral adiposity lymphoma”, “obesity lymphoma”, “body composition lymphoma”. Articles aimed at demonstrating the relationship between abdominal adipose tissue compartments with the CT approach and lymphoma were selected. The titles and abstracts of articles with potential relevance to our topic were analyzed without specific exclusion criteria. The reference list of each article was also carefully analyzed to search for articles of potential relevance to the topic of the present manuscript. After this assessment, we filtered the literature search and selected 7 original articles matching the topic of interest.

## 3. Abdominal Adipose Tissue Quantification and Distribution in Patients with Lymphoma

Cancer cachexia is defined as definite involuntary weight loss due to loss of muscle mass and/or loss of fat tissue. Karmali et al. evaluated cancer cachexia in 86 patients with aggressive B-cell non-Hodgkin lymphoma (B-NHL). The log-rank test and Cox proportional hazards regression were used to investigate the potential impact of cachexia factors on progression-free survival and overall survival. Abdominal muscle mass was quantified with the CT approach on a single slice at the level of the L3 vertebra. The skeletal muscle index was obtained from the cross-sectional muscle area and normalized for height (i.e., skeletal muscle index, cm^2^/m^2^). These data, together with the albumin and the neutrophil-to-lymphocyte ratio, were used to calculate the cancer cachexia index, which allows patients with lymphoma to be classified into two groups: cachectic (*n* = 40) and non-cachectic (*n* = 41). Cachectic patients showed significantly worse progression-free survival (HR 2.18, *p* = 0.044) and overall survival (HR = 4.05, *p* = 0.004) compared to non-cachectic. The results of this study called for improvement of new therapies aimed at preventing cancer cachexia to increase the survival of these patients [17]. Therapeutic strategies for the treatment of cancer cachexia are being developed and include metformin, ghrelin receptor agonists (such as anamorelin), anti-myostatin antibodies and selective androgen receptor modulators (such as GTx-024). Oliveira et al. showed that metformin minimizes tumor-induced wasting by reducing the activity of proteolytic enzymes on muscle [18]. Currently, there are no clinical parameters for the evaluation of cancer cachexia in lymphoma patients that could direct these patients to new therapeutic strategies. The aforementioned study suggests cancer cachexia index as a parameter that could potentially be used to select cachectic patients which could be treated with new therapeutic strategies to improve clinical outcomes [17].

Albano et al. compared high-dose CT (HDCT) and low-dose CT (LDCT) of 18-fludeoxyglucose positron emission tomography (18F-FDG PET)/CT in elderly patients with HL for evaluation of skeletal muscle area (SMA), VAT, SAT and intramuscular adipose tissue (IMAT) area at the abdominal level [19]. Ninety patients were enrolled in the study. Measurements between HDCT and LDCT were compared using Bland–Altman plots and Passing–Bablock regression analyses. The correlation of measurements between HDCT and LDCT was performed by Pearson correlation coefficient (r). Among the parameters compared, a strong correlation was found for VAT (r = 0.942, *p* < 0.0001), for SAT (r = 0.894, *p* < 0.0001) and for SMA (r = 0.934, *p* < 0.0001). The mean difference between these two imaging modalities was small: 1% for SAT, +6.1% for VAT, +2.5% for SMA and −1.9% for IMAT. The authors concluded that LDCT can be considered a precise and accurate method for the quantification of VAT, SAT and SMA. This study found some markedly different measurements for IMAT between HDCT and LDCT. This result can be explained by the low amount of adipose tissue present between the muscle fibers. Furthermore, the segmentation of this compartment of adipose tissue shows greater challenges from an anatomical standpoint compared to SAT and VAT, since it is located between the muscle fibers, and it is necessary to segment numerous tiny and intricate areas of adipose tissue infiltration. Conversely, VAT and the SAT are often more easily segmented, and therefore results seem to be more robust. Furthermore, this body composition analysis used both unenhanced CT images and contrast-enhanced CT images, revealing a high correlation between the two and no significant impact of contrast injection on segmentation of adipose and muscle tissues [19].

Albano et al. evaluated body composition changes after treatment in elderly patients with HL (>65 years) using PET/CT. Eighty-eight patients (47 female) were included in the study. The most frequent histological subtype was nodular sclerosis (46 patients), followed by mixed cellularity (23 patients), lymphocyte rich (5 patients), lymphocyte depletion (4 patients) and not specified (10 patients). Staging according to the Ann Arbor System divided 22 patients and 66 patients into early stage (stages I and II) and advanced stage (stages III and IV), respectively. This study demonstrated a significant decrease in muscular areas during treatment (*p* < 0.001) and a significant increase in adipose tissue compartments (i.e., SAT, VAT, IMAT and TAT) over this time (*p* < 0.001) [20]. Different subtypes of HL were included in this study without assessing adipose tissue distribution according to the different histotypes at baseline. Further studies will be important to quantify adipose tissue compartments in the different HL histological subtypes.

Lucijanic et al. evaluated perirenal adipose tissue thickness and SAT thickness in 82 newly diagnosed HL patients [21]. The quantification of the adipose tissue compartments was performed by measuring the greatest and the smallest distance from the contour of the renal parenchyma to the abdominal wall at the level of the renal vein. SAT thickness was evaluated by measuring the distance between the skin and the abdominal wall, at the level of the umbilicus. Significant differences were found according to the European Organization for Research and Treatment of Cancer disease stage; patients with more advanced disease had a higher minimum thickness of perirenal adipose tissue and a lower thickness of SAT (both *p* < 0.05). Similar results were found with the International Prognostic Score stage disease for perirenal adipose tissue (r = 0.34, *p* = 0.002) and SAT (r = −0.27, *p* = 0.013). Furthermore, an optimal cut-off was defined by ROC curve analysis points survival for minimal perirenal adipose tissue thickness (>2 mm; 33/82 (40.2%) patients), maximal perirenal adipose tissue thickness (>25 mm; 29/82 (35.4%) patients) and SAT thickness (≤22 mm; 54/82 (65.9%) patients). Univariate analysis showed that higher minimal perirenal adipose tissue thickness (hazard ratio (HR) = 8.4; *p* < 0.001), higher maximal perirenal adipose tissue thickness (HR = 3.15; *p* = 0.049) and lower SAT thickness (HR = 3.57; *p* = 0.033) were significantly associated with inferior overall survival. This study demonstrates that a lower amount of SAT and a higher amount of perirenal adipose tissue correlate with more advanced disease features and shorter survival in HL patients. Moreover, a lower SAT was associated with faster disease progression. This study demonstrates how quantitative assessment of body composition can be of value, also taking into account that for the same BMI two subjects might have a different tissue distribution in terms of SAT or perirenal adipose tissue (which is part of VAT), with different metabolic profiles. Indeed, the compartments of adipose tissue influence the progression of HL differently [21].

Hinnerichs et al. evaluated the prognostic role of VAT and SAT in 74 patients with primary central nervous system lymphoma (PCNSL) [22]. Quantification of VAT and SAT was performed on the axial plane on CT images at L3 vertebra level. The VAT/SAT ratio was then calculated. This study demonstrated no correlation between adipose tissue parameters measured with either overall survival or progression-free survival in PCNSL patients, suggesting that these parameters cannot be used as reliable biomarkers for PCNSL [22].

Camus et al. evaluated body composition in DLBCL elderly patients treated with immunochemotherapy (i.e., chemotherapy and rituximab). The cachexia score, obtained by quantification of skeletal muscle and abdominal adipose tissue compartments (i.e., VAT and SAT) assessed by CT scan on single slice at L3 vertebra level, was used. Sarcopenia was found in 44 patients included in the study, while 46 patients were found to be adipopenic. The median progression-free survival was 13.6 months in the adipopenic group and 49.4 months in the non-adipopenic group (HR = 2.27; 95% confidence interval (CI): 1.3–4; *p* = 0.0042). The median overall survival was 25.7 months in the adipopenic group and 57.1 months in the non-adipopenic group (HR = 1.93; 95% CI: 1.05–3.55; *p* = 0.0342). Multivariate analysis demonstrated that cachexia scores including sarcopenia and adipopenia were predictive for prognosis independently of BMI and the international prognostic index. This study demonstrated that sarcopenia and adipopenia are predictive of overall survival, while they are not predictive of progression-free survival in multivariate analysis, suggesting that sarcopenia and adipopenia are mostly associated with physiological status rather than disease-specific effects of lymphoma. Body composition analysis performed on CT images demonstrated that cachexia is a reversible process that should be treated with specific therapeutic agents in addition to lymphoma standard therapies [23].

**Figure 1:**
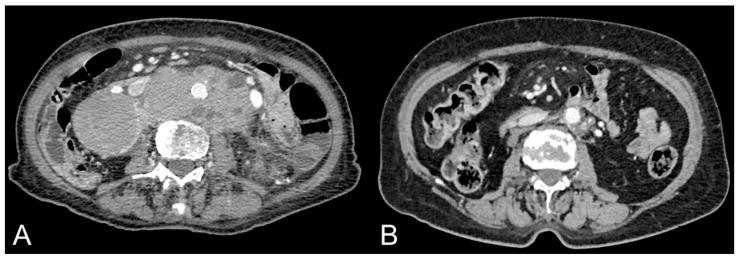
Contrast-enhanced CT images of a 84-year-old female patient with lymphoma showing mass-like abdominal lymphadenopaties (**A**). Note the complete response after treatment, with resolution of the mass together with increase in VAT and SAT (**B**).

Xiao et al. evaluated longitudinal body composition changes in 343 DLBCL patients treated with cyclophosphamide, doxorubicin, vincristine and prednisone, with or without rituximab, and identified clinical variables related to development of sarcopenia and visceral obesity [24]. Almost all patients were male (96.8%) and Caucasian (87.1%). Moreover, among patients, 42.4% had stage I/II, 57% had stage III/IV while 0.6% had unknown staging. VAT, SAT and skeletal muscle areas were quantified using CT images. Repeated-measures analysis of variance and logistic regression was used for data analysis. During the treatment, a decrease in skeletal muscle area was detected, which then returned to baseline 24 months after treatment. SAT increased from baseline of 6.5% during therapy (95% CI = 2.6% to 10.5%) and of 21.4% by 24 months after therapy (95% CI = 15.7% to 27.2%). VAT increased from baseline of 4.5% during therapy (95% CI = −0.9% to 9.9%) and of 21.6% at 24 months after therapy (95% CI = 14.8% to 28.4%). This study demonstrated that DCLBL patients exhibit unfavorable long-term body composition changes also associated with therapies (Figure 1) [24]. This study does not provide data on the different histological subtypes of DCLBL. Further studies should evaluate the distribution of abdominal adipose tissue compartments according to histological subtypes of DCLBL to evaluate the relationship of these tissues on pathogenesis, response to therapies and prognosis.

Wadhwa et al. evaluated body composition in association with chemotherapy toxicity (i.e., chemotoxicity) in children with HL (*n* = 45), NHL (*n* = 42) and rhabdomyosarcoma (*n* = 20) [25]. Body composition was examined using the skeletal muscle index, skeletal muscle density and height-adjusted TAT. Hematologic toxicities were assessed as grade 4 or higher hematologic toxicities and grade 3 or higher non-hematologic toxicities within 6 months since the diagnosis. The median skeletal muscle index was 41.0 cm^2/^m^2^ (range, 25.8–68.6 cm^2^/m^2^), the median skeletal muscle density was 54.1 HU (range, 35–69.4 HU) and the median height-adjusted TAT was 19.5 cm^2^/m^2^ (range, 0–226.7 cm^2^/m^2^). Only the skeletal muscle density at the diagnosis was associated with lower odds of grade 4 or higher hematologic toxicity, whereas skeletal muscle index and height-adjusted TAT were not associated with hematologic toxicities. Furthermore, body composition did not show any association with non-hematologic toxicity [25].

Tram et al. used CT imaging to evaluate body composition changes in pediatric, adolescent and young adult patients with lymphoma [26]. This retrospective analysis was performed by quantifying the SAT, VAT and skeletal muscle at the level of the L3 vertebra using five consecutive axial CT images. The results showed a significant increase in SAT and VAT and a significant decrease in skeletal muscle. BMI% showed no change from baseline to the first follow-up and did not correlate with modifications of the amount of fat and muscle tissue detected on CT images. Male patients with NHL (stage 3 or 4 disease) younger than 12 years of age showed significantly greater adipose tissue increase and significant skeletal muscle decrease after the first treatment cycle. The observed changes in body composition may offer clues towards personalized treatment approaches in pediatric and young adult patients with lymphoma [26].

Taken together, published studies on the topic highlight the importance of assessing body composition in patients with lymphoma. Data and results of individual clinical studies are summarized in Table 1.

BMI does not provide data on the quantification of each compartment of fat tissue or muscle mass. Body composition imaging, with CT or magnetic resonance imaging (MRI), is of fundamental importance for evaluating the relationship that each soft tissue compartment might have with development, progression and prognosis of lymphoma. Most of the studies analyzed quantified these tissues (i.e., abdominal fat or muscles) on a single slice at the level of the L3 vertebra; just one study used five consecutive axial CT images at the level of the L3 vertebra, while one study performed body composition analysis by measuring the thicknesses of the SAT and perirenal adipose tissue. Cachectic lymphoma patients showed a lower overall survival, suggesting that additional therapies should be integrated in this category of patients to improve survival. Furthermore, the effect of immunochemotherapy on the body composition was evaluated, reporting increase in fat mass and decrease in lean mass. As regards hematological toxicity, an association was found with skeletal muscle density but not with TAT.

Lifestyle and nutrition are fundamental to the quality of life in NHL survivors. Indeed, in this category of patients, an unhealthy lifestyle including unbalanced diet, sedentary lifestyle, smoking and body composition change can lead to cancer-treatment-induced metabolic syndrome with abdominal obesity, sarcopenia and insulin resistance. Daniele et al. evaluated how unhealthy lifestyle, types of anti-cancer treatment and body composition changes may increase the risk of metabolic syndrome and sarcopenia [27]. Sixty NHL patients (26 males and 34 females, median age of 46.5, range 22–82) in continuous remission for at least 3 years were included in the study. Fifty-seven patients had the DLBCL histology, and three patients had follicular lymphoma grade III. All patients were treated with R-CHOP, CHOP or CHOP-like treatments including high-dose steroids. Weight gain and related body composition changes were detected in 60% of patients experiencing metabolic syndrome with a higher waist circumference value than patients without metabolic syndrome in both women and men. In this respect, the univariate analysis found a significantly higher risk of metabolic syndrome in patients with an unhealthy lifestyle and in patients who underwent treatment with steroids [27].

Imaging methods such as CT or MRI are the best tools for non-invasive tissue evaluation. More advanced imaging post-processing techniques, such as texture analysis and artificial intelligence, are additional perspectives for precision medicine to be combined with the assessment of body composition metrics and other data. For example, the correlation between body fat at a young age (i.e., childhood, adolescence and young adulthood; age ≤ 30 years) and DLBCL is known [28]. Consequently, not only is the disease evaluated but also the patient as a whole to obtain additional information in an opportunistic way with minimal, if any, additional cost to improve and tailor personalized therapies, possibly improving clinical outcomes [29,30,31,32,33,34]. Indeed, artificial intelligence is a revolutionary technology that can obtain an automatic, fast and precise analysis of tissues [33,34].

Moreover, radiomics can obtain predictive data in medical imaging [30]. This field is based on radiological images to obtain quantitative data that can predict features including tumor biology, molecular patterns or immune cell infiltrates. The potential advantages would be multiple: evaluation of the intratumoral heterogeneity and the interactions with the microenvironment, analysis of multiple lesions in parallel, evaluation of the variations of the lesions longitudinally over time, and, lastly, the non-invasiveness of the technique [32].

It would be interesting to apply radiomics and artificial intelligence to evaluate the abdominal adipose tissue in patients with lymphoma in further studies.

## 4. Conclusions

CT-based quantification and distribution of abdominal adipose tissue can be relevant for the relationship with clinical outcomes in patients with lymphoma. The segmentation of the adipose tissue compartments performed at the first diagnosis and follow-up can be useful to prevent the cachectic state or make it reversible through the use of specific therapeutic agents under development, towards the concept of precision medicine. Further studies should be performed to evaluate the effect of new therapeutic agents on body composition to anticipate and treat cancer cachexia in patients with lymphoma. Furthermore, considering the effect of lymphoma therapies on adipose tissue (e.g., increase in both SAT and VAT), further studies should be conducted to evaluate the impact of targeted diets and lifestyle interventions to tackle visceral obesity in patients with lymphoma under therapy. Artificial intelligence and radiomics are interesting and novel post-processing techniques that can be applied to the opportunistic analysis of CT or MR imaging, possibly improving the current standard of care in patients with lymphoma.

## Figures and Tables

**Table 1:** Summary of CT studies evaluating quantity and distribution of abdominal adipose tissue in patients with lymphoma.

Authors	Purpose	Adipose Tissue Compartments	Number of Patients	Results
Albano et al. (2021) [19]	To compare HDCT and LDCT of 18F-FDG PET)/CT in elderly patients with HL for evaluation of abdominal adipose tissue compartments in HL patients	VAT SAT IMAT	90	VAT (r = 0.942, *p* < 0.0001) SAT (r = 0.894, *p* < 0.0001) IMAT: good correlation but less significant (r = 0.742)
Lucijanic et al. (2021) [20]	To evaluate relationship between abdominal adipose tissue compartments and clinical outcomes in HL patients	Perirenal adipose tissue SAT	82	Higher minimum thickness of perirenal adipose tissue and a lower thickness of SAT (both *p* < 0.05) in patients with advanced disease International Prognostic Score stage disease for perirenal adipose tissue (Rho = 0.34, *p* = 0.002) and SAT (Rho = −0.27, *p* = 0.013) ROC curve analysis points for survival for minimal perirenal adipose tissue thickness (>2 mm; 33/82 (40.2%) patients), maximal perirenal adipose tissue thickness (>25 mm; 29/82 (35.4%) patients) and SAT thickness (≤22 mm; 54/82 (65.9%) patients) Univariate analysis showed higher minimal perirenal adipose tissue thickness (HR = 8.4; *p* < 0.001), higher maximal perirenal adipose tissue thickness (HR = 3.15; *p* = 0.049) and lower SAT thickness (HR = 3.57; *p* = 0.033) were significantly associated with inferior OS
Hinnerichs et al. (2022) [21]	To evaluate relationship between abdominal adipose tissue compartments and clinical outcomes in PCNSL patients	VAT SAT	74	No correlations
Camus et al. (2014) [22]	To evaluate body composition in elderly patients treated with immunochemotherapy in DLBCL patients	VAT SAT	90	The median PFS was 13.6 months in the adipopenic group and 49.4 months in the non-adipopenic group (hazard ratio (HR) = 2.27; 95% confidence interval (CI): 1.3–4; *p* = 0.0042) The median OS was 25.7 months in the adipopenic group and 57.1 months in the non-adipopenic group (HR = 1.93; 95% CI: 1.05–3.55; *p* = 0.0342)
Xiao et al. (2016) [23]	To evaluate longitudinal body composition changes and identified clinical variables related with development of sarcopenia and visceral obesity in DLBCL patients	VAT SAT	343	SAT increased from baseline of 6.5% during therapy (95% confidence interval (CI) = 2.6% to 10.5%) and of 21.4% by 24 months after therapy (95% CI = 15.7% to 27.2%) VAT increased from baseline of 4.5% during therapy (95% CI = −0.9% to 9.9%) and of 21.6% by 24 months after therapy (95% CI = 14.8% to 28.4%)
Wadhwa et al. (2022) [24]	To evaluate body composition in association with chemotherapy toxicity in patients with HL, NHL and rhabdomyosarcoma	height-adjusted TAT	107	No correlations
Tram et al. (2022) [25]	To evaluate body composition changes in pediatric, adolescent and young adult patients with lymphoma	VAT SAT	110	Male patients with NHL with stage 3 or 4 disease younger than 12 years of age showed significantly greater adipose tissue after the first treatment cycle

18F-FDG PET, 18-fludeoxyglucose positron emission tomography; CT, computed tomography; DLBCL, diffuse large B-cell lymphoma; HDCT, high-dose computed tomography; HL, Hodgkin lymphoma; IMAT, intramuscular adipose tissue; LDCT, low-dose computed tomography; NHL, non-Hodgkin lymphoma; PCNSL, primary central nervous system lymphoma; OS, overall survival; PFS, progression-free survival; SAT, subcutaneous adipose tissue; TAT, total adipose tissue; VAT, visceral adipose tissue.

## Data Availability

Not applicable.

## References

[1] Louie S.M., Roberts L.S., Nomura D.K. (2013). Mechanisms linking obesity and cancer. Biochim. Biophys. Acta.

[2] Larsson S.C., Wolk A. (2011). Body mass index and risk of non-Hodgkin’s and Hodgkin’s lymphoma: A meta-analysis of prospective studies. Eur. J. Cancer.

[3] Murphy F., Kroll M.E., Pirie K., Reeves G., Green J., Beral V. (2013). Body size in relation to incidence of subtypes of haematological malignancy in the prospective Million Women Study. Br. J. Cancer.

[4] Lichtman M.A. (2010). Obesity and the risk for a hematological malignancy: Leukemia, lymphoma, or myeloma. Oncologist.

[5] Matos A., Marinho-Dias J., Ramalheira S., Oliveira M.J., Bicho M., Ribeiro R. (2016). Mechanisms underlying the association between obesity and Hodgkin lymphoma. Tumor Biol..

[6] Carlsen H., Haugen F., Zadelaar S., Kleemann R., Kooistra T., Drevon C.A., Blomhoff R. (2009). Diet-induced obesity increases NF-kappaB signaling in reporter mice. Genes Nutr..

[7] Jost P.J., Ruland J. (2007). Aberrant NF-kappaB signaling in lymphoma: Mechanisms, consequences, and therapeutic implications. Blood.

[8] Nagel D., Vincendeau M., Eitelhuber A.C., Krappmann D. (2014). Mechanisms and consequences of constitutive NF-kappaB activation in B-cell lymphoid malignancies. Oncogene.

[9] Jiménez-Cortegana C., Hontecillas-Prieto L., García-Domínguez D.J., Zapata F., Palazón-Carrión N., Sánchez-León M.L., Tami M., Pérez-Pérez A., Sánchez-Jiménez F., Vilariño-García T. (2022). Obesity and Risk for Lymphoma: Possible Role of Leptin. Int. J. Mol. Sci..

[10] Park Y.H., Lee J.K., Kim K.M., Kook H.R., Lee H., Kim K.B., Lee S., Byun S.S., Lee S.E. (2014). Visceral obesity in predicting oncologic outcomes of localized renal cell carcinoma. J. Urol..

[11] Ibrahim M.M. (2010). Subcutaneous and visceral adipose tissue: Structural and functional differences. Obes. Rev..

[12] Despres J.P., Lemieux I. (2006). Abdominal obesity and metabolic syndrome. Nature.

[13] Zhang H.P., Zou J., Xu Z.Q., Ruan J., Yang S.D., Yin Y., Mu H.J. (2017). Association of leptin, visfatin, apelin, resistin and adiponectin with clear cell renal cell carcinoma. Oncol. Lett..

[14] Mallio C.A., Greco F., Pacella G., Schena E., Beomonte Zobel B. (2018). Gender-based differences of abdominal adipose tissue distribution in non-small cell lung cancer patients. Shanghai Chest.

[15] Greco F., Cirimele V., Mallio C.A., Beomonte Zobel B., Grasso R.F. (2018). Increased visceral adipose tissue in male patients with clear cell renal cell carcinoma. Clin. Cancer Investig. J..

[16] Greco F., Mallio C.A. (2021). Relationship between visceral adipose tissue and genetic mutations (VHL and KDM5C) in clear cell renal cell carcinoma. Radiol. Med..

[17] Karmali R., Alrifai T., Fughhi I.A.M., Ng R., Chukkapalli V., Shah P., Basu S., Nathan S., Szymanski-Grant K., Gordon L.I. (2017). Impact of cachexia on outcomes in aggressive lymphomas. Ann. Hematol..

[18] Oliveira A.G., Gomes-Marcondes M.C. (2016). Metformin treatment modulates the tumour-induced wasting effects in muscle protein metabolism minimising the cachexia in tumour-bearing rats. BMC Cancer.

[19] Albano D., Camoni L., Rinaldi R., Tucci A., Zilioli V.R., Muzi C., Ravanelli M., Farina D., Coppola A., Camalori M. (2021). Comparison between skeletal muscle and adipose tissue measurements with high-dose CT and low-dose attenuation correction CT of ^18^F-FDG PET/CT in elderly Hodgkin lymphoma patients: A two-centre validation. Br. J. Radiol..

[20] Albano D., Dondi F., Treglia G., Tucci A., Ravanelli M., Farina D., Bertagna F. (2022). Longitudinal Body Composition Changes Detected by [18F]FDG PET/CT during and after Chemotherapy and Their Prognostic Role in Elderly Hodgkin Lymphoma. Cancers.

[21] Lucijanic M., Huzjan Korunic R., Ivic M., Fazlic Dzankic A., Kusec R., Pejsa V. (2021). Perirenal and subcutaneous fat differently affect outcomes in newly diagnosed classical Hodgkin lymphoma patients. Hematol. Oncol..

[22] Hinnerichs M., Ferraro V., Zeremski V., Mougiakakos D., Omari J., Pech M., Bär C., Wienke A., Saalfeld S., Strobel A. (2022). Prognostic Impact of Quality and Distribution of Adipose Tissue in Patients with Primary Central Nervous System Lymphoma. Vivo.

[23] Camus V., Lanic H., Kraut J., Modzelewski R., Clatot F., Picquenot J.M., Contentin N., Lenain P., Groza L., Lemasle E. (2014). Prognostic impact of fat tissue loss and cachexia assessed by computed tomography scan in elderly patients with diffuse large B-cell lymphoma treated with immunochemotherapy. Eur. J. Haematol..

[24] Xiao D.Y., Luo S., O'Brian K., Sanfilippo K.M., Ganti A., Riedell P., Lynch R.C., Liu W., Kahl B.S., Cashen A.F. (2016). Longitudinal Body Composition Changes in Diffuse Large B-cell Lymphoma Survivors: A Retrospective Cohort Study of United States Veterans. J. Natl. Cancer Inst..

[25] Wadhwa A., Adams K.M., Dai C., Richman J.S., McDonald A.M., Williams G.R., Bhatia S. (2022). Association between body composition and chemotherapy-related toxicity in children with lymphoma and rhabdomyosarcoma. Cancer.

[26] Tram N.K., Chou T.H., Ettefagh L.N., Deep K., Bobbey A.J., Audino A.N., Stacy M.R. (2022). Quantification of chemotherapy-induced changes in body composition in pediatric, adolescent, and young adult lymphoma using standard of care CT imaging. Eur. Radiol..

[27] Daniele A., Guarini A., Summa S.D., Dellino M., Lerario G., Ciavarella S., Ditonno P., Paradiso A.V., Divella R., Casamassima P. (2021). Body Composition Change, Unhealthy Lifestyles and Steroid Treatment as Predictor of Metabolic Risk in Non-Hodgkin’s Lymphoma Survivors. J. Pers. Med..

[28] Hidayat K., Du X., Shi B.M. (2018). Body fatness at a young age and risks of eight types of cancer: Systematic review and meta-analysis of observational studies. Obes. Rev..

[29] Mallio C.A., Napolitano A., Castiello G., Giordano F.M., D’Alessio P., Iozzino M., Sun Y., Angeletti S., Russano M., Santini D. (2021). Deep Learning Algorithm Trained with COVID-19 Pneumonia Also Identifies Immune Checkpoint Inhibitor Therapy-Related Pneumonitis. Cancers.

[30] Marano M., Vespasiani Gentilucci U., Altamura C., Siotto M., Squitti R., Bucossi S., Quintiliani L., Migliore S., Greco F., Scarciolla L. (2015). Altered metal metabolism in patients with HCV-related cirrhosis and hepatic encephalopathy. Metab. Brain Dis..

[31] Lambin P., Rios-Velazquez E., Leijenaar R., Carvalho S., van Stiphout R.G., Granton P., Zegers C.M., Gillies R., Boellard R., Dekker A. (2012). Radiomics: Extracting more information from medical images using advanced feature analysis. Eur. J. Cancer.

[32] Malone E.R., Sim H.W., Stundzia A., Pierre S., Metser U., O'Malley M., Sacher A.G., Sridhar S.S., Hansen A.R. (2022). Predictive radiomics signature for treatment response to nivolumab in patients with advanced renal cell carcinoma. Can. Urol. Assoc. J..

[33] Greco F., Mallio C.A. (2021). Artificial intelligence and abdominal adipose tissue analysis: A literature review. Quant. Imaging Med. Surg..

[34] Greco F., Salgado R., Van Hecke W., Del Buono R., Parizel P.M., Mallio C.A. (2022). Epicardial and pericardial fat analysis on CT images and artificial intelligence: A literature review. Quant. Imaging Med. Surg..

